# Can we revolutionize diagnostic imaging by keeping Pandora’s box closed?

**DOI:** 10.1259/bjr.20230505

**Published:** 2023-10-20

**Authors:** Thomas C Kwee, Derya Yakar, Tim E Sluijter, Jan P Pennings, Christian Roest

**Affiliations:** 1 Department of Radiology, University Medical Center Groningen, University of Groningen, Groningen, Netherlands

## Abstract

Incidental imaging findings are a considerable health problem, because they generally result in low-value and potentially harmful care. Healthcare professionals struggle how to deal with them, because once detected they can usually not be ignored. In this opinion article, we first reflect on current practice, and then propose and discuss a new potential strategy to pre-emptively tackle incidental findings. The core principle of this concept is to keep the proverbial Pandora’s box closed, *i.e*. to not visualize incidental findings, which can be achieved using deep learning algorithms. This concept may have profound implications for diagnostic imaging.

## Introduction

**Figure 1.**
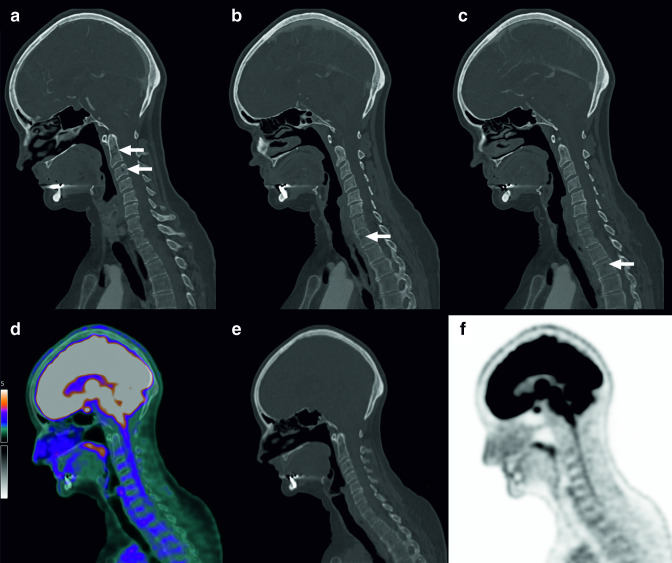
A 62-year-old female presented with suspected acute ischemic stroke, for which CTA of the head and neck was ordered to identify large vessel occlusions suitable for endovascular thrombectomy. CTA angiography was negative for large vessel occlusions, but did show a few hypodense foci of unclear nature in vertebrae C2, C3, T1, and T3 (a–c, arrows) as incidental findings. FDG PET/CT was ordered to evaluate the spine for metastatic disease and to search for a primary tumor. However, FDG-PET/CT did not show any pathology (FDG-PET/CT (d), low-dose CT (e), and FDG-PET (f) images at the level of the cervical—upper thoracic spine are shown). Follow-up MRI 6 months later did not show any pathology either. Therefore, the lesions were classified as benign. CTA, CT angiography; FDG, ^18^F-fluoro-2-deoxy-D-glucose; PET, positron emission tomography.

**Figure 2.**
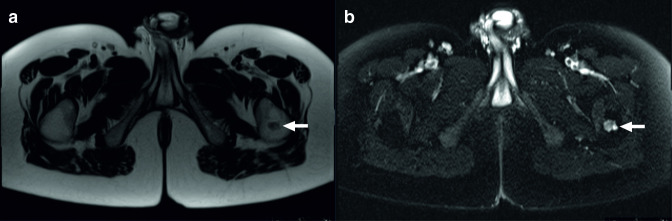
A 30-year-old female with a history of ulcerative colitis and colectomy with ileal pouch-anal anastomosis underwent MRI of the pelvis to evaluate the anastomosis (J-pouch) because of abdominal complaints. At the edge of the scan, a lesion at the left trochanteric region was see as an incidental finding. The 2 cm lobulated intramedullary lesion, visible with low intensity on *T*_1_ weighted imaging (a, arrow) and high signal intensity on fat-suppressed *T*_2_ weighted imaging (b, arrow), was diagnosed as a chondroid tumor based on MRI findings. The patient was referred to an oncologic orthopedic surgeon. The lesion remained asymptomatic and two additional MRI scans did not show any changes of the lesion either during a follow-up of 2 years, indicating benign behavior.

**Figure 3.**
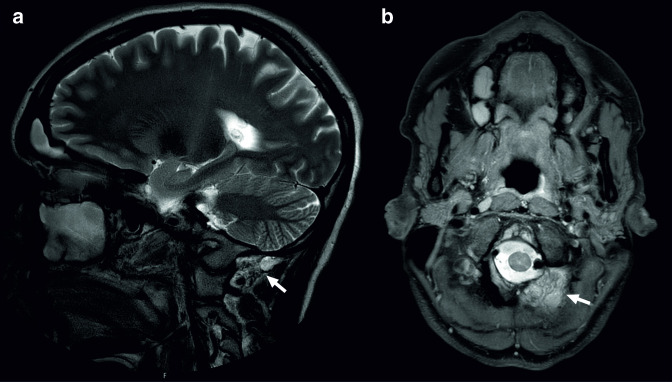
A 32-year-old female presented with frequent headaches, for which MRI of the brain was ordered to exclude any underlying pathology. MRI showed no intracranial pathology, but only signs of sinusitis. MRI also showed a soft-tissue lesion below the left occipital bone, adjacent to the left vertebral artery, as shown on *T*_2_ weighted images (a and b, arrows). The patient was referred to an oncologic surgeon, because soft-tissue sarcoma was in the differential diagnosis. Ultrasound-guided biopsy was performed, which was inconclusive. A second ultrasound-guided biopsy showed a (benign) lymphangioma.

**Figure 4.**
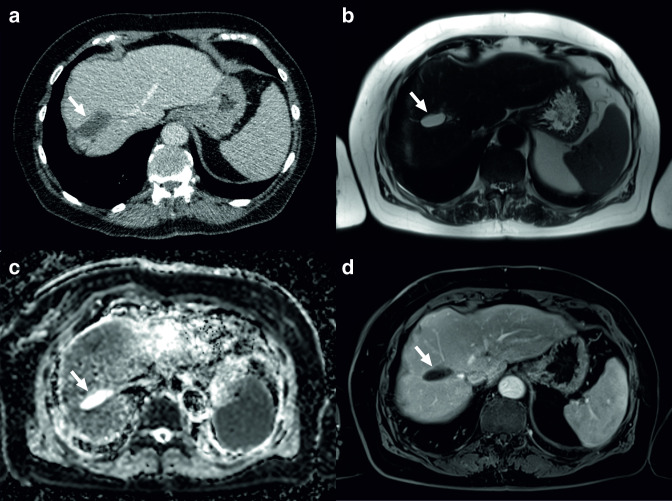
A 72-year-old female underwent CT of the chest because of respiratory symptoms. CT was negative for intrathoracic pathology, but did show a hypodense lesion of unclear nature in the liver (a, arrow) as an incidental finding. MRI showed the lesion to be hyperintense on *T*_2_ weighted imaging (b, arrow), without an impeded diffusion on the apparent diffusion coefficient map (c), and without any enhancement on contrast-enhanced *T*_1_ weighted imaging (d, arrow). The patient was referred to a tertiary care hepatobiliary center, because malignancy could not be excluded. The patient underwent two ultrasound-guided tissue samplings (which were inconclusive), underwent one hepatobiliary scintigraphy (which was inconclusive), and four additional MRI scans. Because the lesion remained unchanged after 3 years of follow-up, it was considered benign.

Incidental imaging findings (*i.e.* imaging findings serendipitously diagnosed in a patient undergoing imaging for an unrelated reason,^1^ later on simply referred to as “incidental findings”) are very common. A few representative examples from routine clinical practice are shown in Figures 1–4. In a meta-analysis on this topic, the mean overall frequency of incidental findings was reported to be 23.6%, with a higher frequency for studies involving CT than other imaging modalities (radiography, ultrasonography, MRI, and positron emission tomography [PET]).^2^ The latter is due to the fact that applied scan volumes for CT are frequently large and CT provides a lot of cross-sectional anatomic information.^3^ Experts have mentioned that most incidentally detected findings are unlikely to be clinically relevant.^4,5^ Incidental findings are analogous to the results of screening tests when screening is applied to unselected, low-risk patients, and they generally result in low-value and potentially harmful care.^5^ Because of advancing technology and the increasing use of cross-sectional imaging (CT, MRI, PET]),^6^ the frequency of incidental findings will continue to increase, along with their economic burden on the healthcare system.^5,7^ An unresolved conundrum is how to best manage incidental findings. In this opinion article, we first reflect on current practice, and then propose and discuss a new potential strategy to pre-emptively tackle incidental findings.

## Current radiology practice: untargeted screening

Radiologists vet clinical requests for imaging and decide on the best imaging modality to answer the question, whilst minimizing radiation and cost. Importantly, imaging examinations very frequently capture many more anatomic structures than actually necessary to answer a specific clinical question. Radiologists are screening all of these “unrequested” anatomic structures for the presence of pathology (Figures 1–4). Such a degree of untargeted screening on such a massive scale (considering that many millions of diagnostic imaging studies are performed worldwide on a daily basis) is not done anywhere else in clinical medicine. Blood tests, which are frequently ordered in daily practice, can be used as an analogy to medical imaging. Numerous different blood parameters can be tested, but there is no single physician who would request all of them on the laboratory request form. This is due to the risk of incidental findings (including many false-positives) when screening for abnormal blood parameters without an appropriate clinical indication. Although individual blood parameters can be easily excluded from testing, unrequested imaging information cannot. It is ”free of charge” and standard of care to routinely screen all anatomic structures that are visualized by an imaging modality. However, the detection of incidental findings is likely to present low-value and potentially harmful care,^5^ or at best non-evidence-based care.

## What about implementing “proven” screening programs in routine patient care?

For breast, colorectal, and lung cancer, some evidence had been reported that screening with imaging (mammography, CT colonography, and low-dose chest CT, respectively) in specific (higher-risk) populations may reduce the risk of dying from these diseases.^8^ Therefore, it can be argued that the detection of an incidental finding on a routine imaging examination in these settings may be useful. For example, if a 60-year-old active smoker with 20 pack-year smoking history were to undergo CT urography because of hematuria, and the CT scan also included the basal lungs, it could be reasoned that it would be worthwhile to detect a lung nodule in the basal lungs, because this patient’s profile matches the selection criteria of the American Cancer Society for lung cancer screening.^9^ However, the criteria of the American Cancer Society also mention that the people who are going to be screened for lung cancer: (1) should receive counseling to quit smoking if they currently smoke, (2) have been told by their doctor about the possible benefits, limits, and harms of screening with CT scans, and (3) can go to a center that has experience in lung cancer screening and treatment.^9^ Neither the referring physician nor the radiologist has assured that these three pre-conditions are fulfilled prior to CT urography. Furthermore, even if patients would be informed about the chance of detecting incidental findings, this would lead to additional issues. For example, imagine a scenario in which a patient undergoes a CT scan of the abdomen for suspected appendicitis, and this patient is informed beforehand that the basal lungs will also be screened for lung cancer. Will we repeat the CT scan if there are too many motion artifacts in the basal lungs? What if the patient asks to screen the entire lungs and not only the basal parts? And if we would decide to screen the entire lungs, would we then inform the patient that we may detect incidental findings in other structures than the lungs at the thoracic level?

## Current management of incidental findings: guidelines

Depending on the clinical context (*e.g.* patient’s age, comorbidities, life expectancy, and patient’s preferences), clinicians and their patients can either ignore incidental findings or choose to actively manage them. There are a few previous studies that have investigated the natural course of incidental findings, *e.g*. for lung nodules,^10^ meningiomas,^11^ and non-functioning pituitary microadenomas,^12^ and several incidental findings guidelines have been published,^13^ which may aid in clinical decision-making. However, for most incidental findings it is simply unknown whether or not they will cause morbidity and/or mortality during a person’s lifetime when left untouched. In addition, there is no evidence that any of the recommendations for additional tests, follow-up or treatment of incidental findings that are given in these guidelines are cost-effective. In fact, if these guidelines were proven cost-effective, then screening for incidental findings would already have been expanded to the general population, which is not the case.

## Can existing incidental findings guidelines be improved when adhered to?

It has been advocated to enrich incidental findings guidelines with a call for scientific studies to confirm that these guidelines are working as intended (*i.e.* to prove they produce high-value care) and to improve them.^5^ Incidental findings may have two outcomes: either they will cause morbidity and/or mortality during a person’s lifetime, or they do not. The clinical relevance of an incidental finding can only be determined when left untouched. However, current incidental findings guidelines frequently recommend a referral for management. For example, incidentally detected solid renal masses ≥1.0 cm, presumed to represent renal cell carcinoma, may be subjected to biopsy and/or surgery or percutaneous ablation.^14^ However, a pathological diagnosis of cancer does not necessarily mean that a lesion would have become clinically relevant.^15^ For example, autopsy studies have shown that the great majority of incidentally discovered renal cell carcinomas have no distant metastases on autopsy.^16–18^ The incidental findings guidelines introduce bias when they lead to tissue sampling and/or treatment (an indolent cancer may be diagnosed and “successfully” treated, although it would never have caused morbidity and/or mortality during a person’s lifetime). As such, incidental findings guidelines can be regarded as an obstacle in defining the true clinical relevance of incidental findings. In order to circumvent this issue, it has been proposed to conduct prospective randomized trials for each type of incidental findings in which deferral of aggressive diagnosis and management is a treatment arm.^5^ However, it remains highly questionable if a sufficiently large and representative sample of patients would be willing to participate in such a trial once they are aware a potentially dangerous lesion is present in their body.

## A potential solution: keep Pandora’s box closed

**Figure 5.**
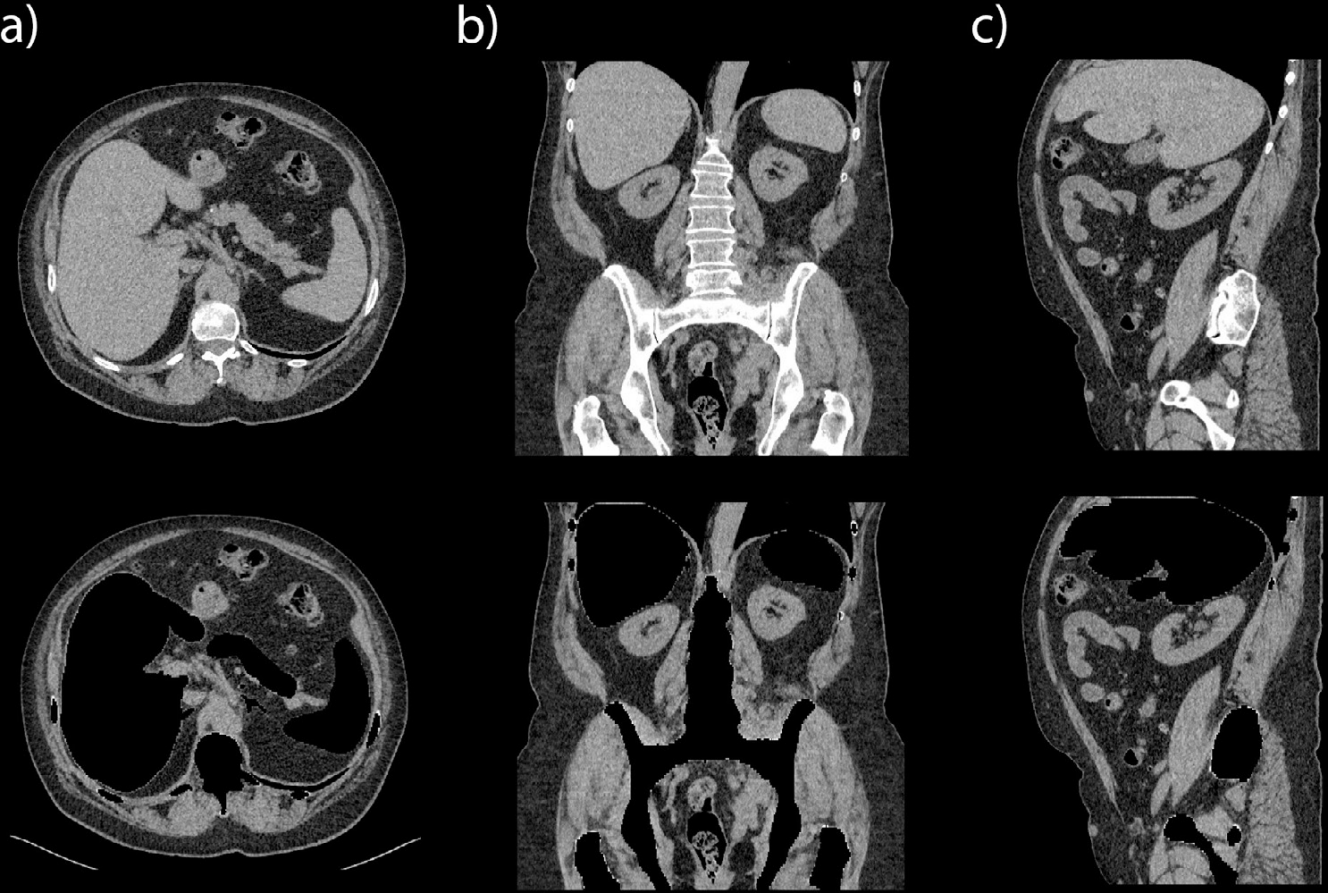
Example of keeping Pandora’s box closed with a primary segmentation of clinically irrelevant organs in unenhanced CT for urolithiasis. Upper panel shows unmodified CT slices in axial (**a**), coronal (**b**), and sagittal (**c**) directions. Lower panel shows corresponding modified CT slices on which several organs that can be considered irrelevant when evaluating for urolithiasis (liver, gallbladder, pancreas, spleen, and bone) have been eliminated (blacked out) using a deep learning algorithm. A corresponding video with axial and coronal slices is shown in Supplementary file 1.

**Figure 6.**
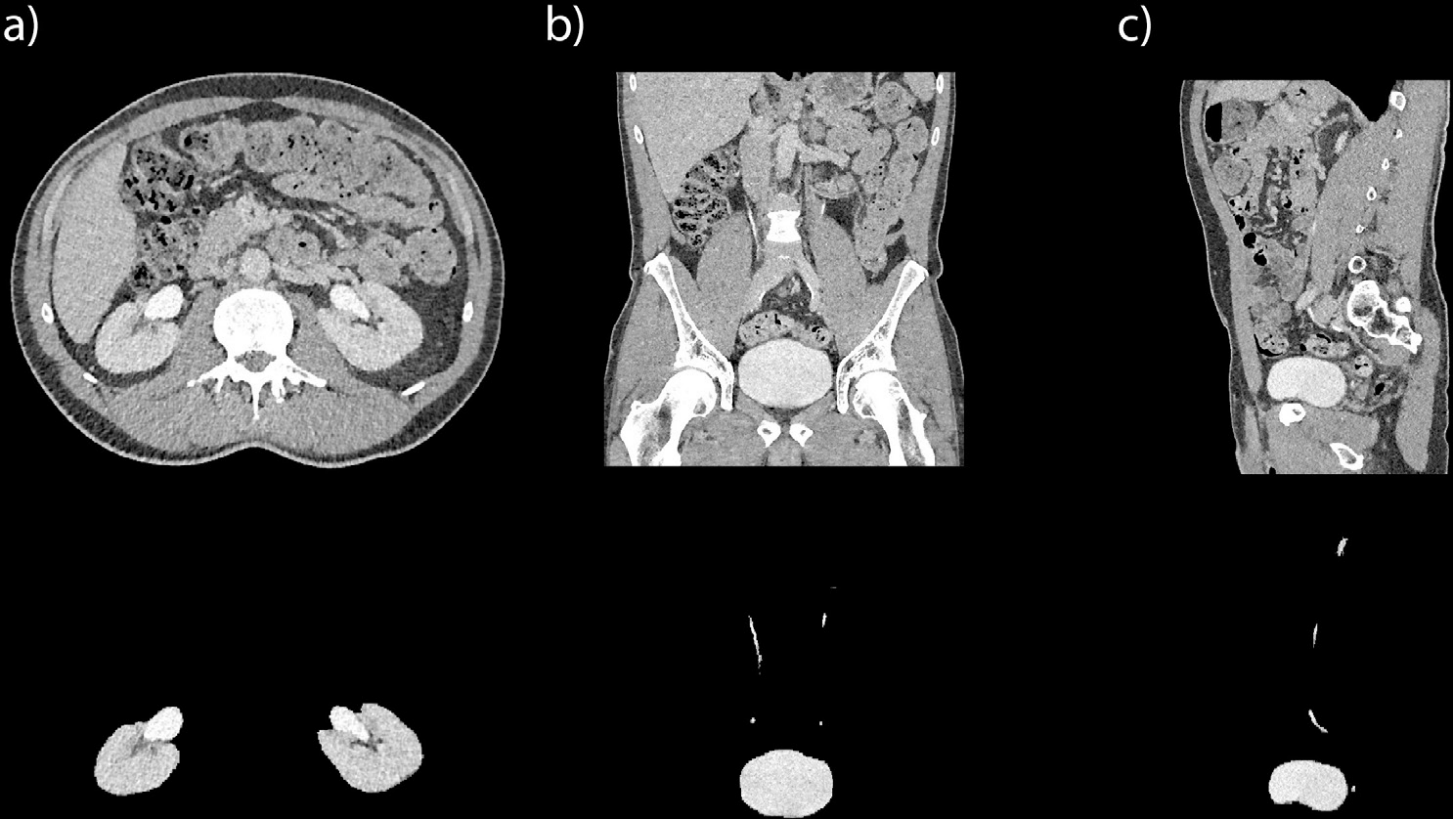
Example of keeping Pandora’s box closed with a primary segmentation of clinically relevant organs in CT urography for hematuria. Upper panel shows unmodified CT slices in axial (**a**), coronal (**b**), and sagittal (**c**) directions. Lower panel shows corresponding modified CT slices on which only the kidneys, ureters, and urinary bladder are visible and all other anatomic structures that can be considered irrelevant when evaluating for hematuria have been eliminated (blacked out) using a deep learning algorithm. A corresponding video with axial and coronal slices is shown in Supplementary file 2.

**Figure 7.**
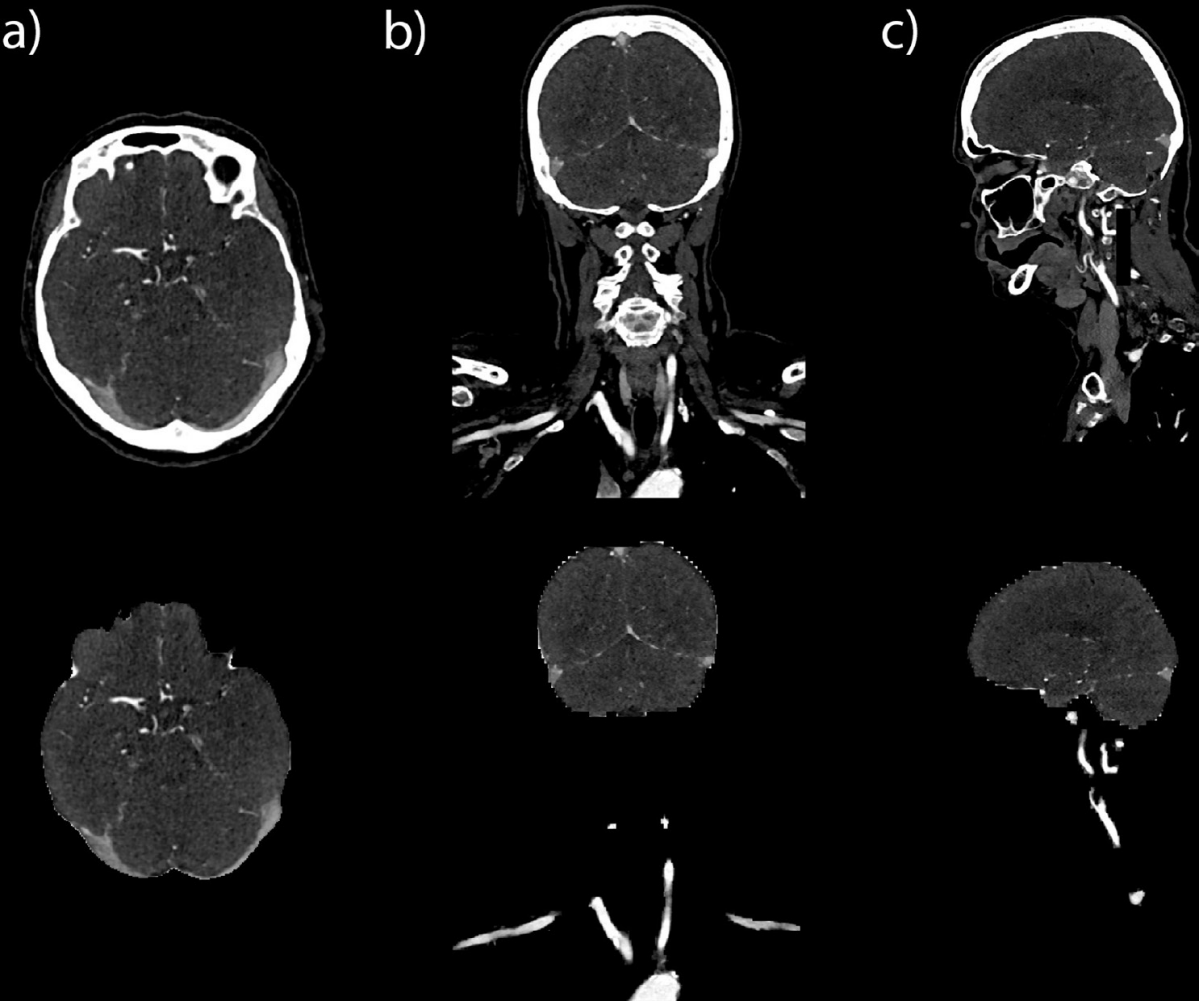
Example of keeping Pandora’s box closed with a primary segmentation of clinically relevant organs in CT angiography for acute ischemic stroke. Upper panel shows unmodified CT slices in axial (**a**), coronal (**b**), and sagittal (**c**) directions. Lower panel shows corresponding modified CT slices on which only the extracranial arteries and brain with intracranial arteries are visible, and all other anatomic structures that can be considered irrelevant when evaluating for large vessel occlusions have been eliminated (blacked out) using a deep learning algorithm. A corresponding video with axial and coronal slices is shown in Supplementary file 3.

A new potential concept to preemptively tackle incidental findings is by keeping the proverbial Pandora’s box closed, *i.e*. to not visualize incidental findings that may result in low-value (or non-evidence-based) and potentially harmful care. This can be achieved by applying deep learning-based organ segmentation algorithms that eliminate organs that may harbor an incidental finding from medical images prior to diagnostic evaluation. Either a primary segmentation of *clinically irrelevant organs* can be done (which will then be deleted from the imaging examination prior to diagnostic evaluation, Figure 5, video in Supplementary file 1) or a primary segmentation of *clinically relevant organs* can be done (which will then be the only organs that are displayed for diagnostic review, Figures 6 and 7, videos in Supplementary files 2 and 3). Recent studies have shown the feasibility of these two segmentation approaches for keeping Pandora’s box closed in CT for urolithiasis and CT angiography for acute ischemic stroke.^19,20^ This approach is analogous to blood tests: numerous parameters can be tested in the blood that is available in the collection tube, but only the clinically relevant parameters are assessed. However, the collected blood is mostly available for some time in the laboratory to test any additional parameters when clinically indicated. Similarly, the unmodified imaging study should be digitally archived but kept hidden from human eyes to avoid accidental detection of an incidental finding. Archiving of the unmodified imaging study is required, because in the future, it may become clinically necessary to evaluate organs that were hidden by the segmentation (Figures 5–7 and 1 [Supplementary-material suppl2]-3).

Supplementary file 1.

Supplementary file 2.

Supplementary file 3.

## Which organs are clinically (ir)relevant?

The distinction between clinically relevant organs *vs* clinically irrelevant organs is dynamic and depends on the clinical questions of the referring physician. Organs that do not need to be visualized to answer the clinical questions of the referring physician can be considered irrelevant. For example, if a neurologist asks for an MRI scan of the cervical spine to evaluate for disc herniations in a patient with signs of cervical radiculopathy, the thyroid gland (which is often in the field of view) can be considered an irrelevant organ. In addition, if an urologist asks for CT urography to screen the upper urinary tract for pathology in a patient with hematuria and a negative cystoscopy, all organs outside the urinary tract can be considered irrelevant unless an upper urinary tract tumor is detected and an assessment for metastatic disease becomes necessary. Note that the vast majority of patients with hematuria do not have any cancer in the upper urinary tract.^21^ Only when an upper urinary tract tumor is detected on CT urography, it can be decided to retrieve the unmodified CT examination from the digital archives for complete staging. At times, it may not be possible to determine with certainty beforehand if an organ is clinically relevant or not. For example, in patients with suspected appendicitis or diverticulitis, many alternative abdominal diseases and even pneumonia may explain the symptoms.^22,23^ In these patients, it may be unwise to delete abdominal organs and basal lungs from the (CT) scan. However, bone could be safely deleted from the (CT) scan in suspected appendicitis or diverticulitis because explanatory pathology is virtually never located there.^22,23^

## Additional advantages of keeping Pandora’s box closed

Keeping Pandora’s box closed may have several other advantages besides hiding incidental findings that may lead to low-value (or non-evidence-based) and potentially harmful care. First, referring physicians have to decide together with radiologists which organs should be visualized and which ones can be deleted from the imaging study. In order to make this decision, the quality of clinical reasoning and the quality of imaging requests have to be of high standards. Note that the quality of clinical reasoning has generally decreased over the past decades,^24–26^ and that the quality of imaging requests has also been reported to be inadequate in the majority of cases.^27^ An improvement of these two parameters will increase the diagnostic value of an imaging study according to the “quality in, quality out” principle. Second, radiologists will be enabled to provide more meaningful healthcare, because they will only focus on those organs that matter to answer the referring physicians questions rather than spending time on unrequested untargeted screening. A more focused image analysis will reduce interpretation time, which may theoretically also decrease the risk of diagnostic errors because less data have to be evaluated.^28–30^ This, in turn, may perhaps decrease the fear among some radiologists for malpractice suits. Third, if the frequency of incidental findings decreases, radiologists will less frequently be asked to perform invasive biopsies and/or imaging follow-up “to exclude malignancy” of incidental findings that are likely benign or clinically irrelevant (Figures 3 and 4). Altogether, the potential advantages for radiology practice may perhaps also increase job satisfaction and decrease the risk of burnout among radiologists.

## Will patients and medical doctors accept a closed Pandora’s box?

The general conception in the population is that screening and early detection of potentially curable lesions (most notably cancer) is beneficial.^31^ Not surprisingly, the vast majority of patients wants all unrequested incidental findings to be found on an imaging study.^32^ Many medical doctors (including radiologists) may have similar thoughts. Interestingly, however, when it comes to whole-body screening with MRI or CT in asymptomatic individuals, there are no professional medical organizations that endorse them, although this is fundamentally not different from untargeted screening of organs for incidental findings on routine clinical imaging studies.

Despite mainstream thinking, the balance may be shifting. In a *New England Journal of Medicine* paper, it was mentioned that “clinicians should withhold information that is likely to overwhelm and distress patients if their having the information would provide no obvious benefit and they don’t ask for it; information overload (especially if the information is not clinically relevant) may render more important discussions (with patients) impossible”.^33^ On another note, some radiologists are already censoring information for referring physicians and patients. For example, the problem of overdiagnosis and overtreatment of low-risk thyroid cancer is well known.^34^ In our hospital (University Medical Center Groningen, Groningen, The Netherlands), many radiologists completely ignore all incidentally visible thyroid nodules on CT performed for other reasons, and do not report them.

Financial incentives may perhaps facilitate the introduction of the proposed concept of keeping Pandora’s box closed. Blood tests can again be used as an analogy: for each blood parameter costs are charged. A similar methodology may be used for medical imaging, *i.e*. to charge costs per organ or organ system that has to be visualized and diagnostically evaluated, and not for those that can be digitally deleted from the imaging study. This will stimulate referring physicians to improve their quality of clinical reasoning and imaging requests, and may potentially reduce healthcare costs. Opponents of keeping Pandora’s box closed may contend that it is not proven cost-effective and ask for scientific evidence, even though the (rhetoric) counter question can be asked: is screening for incidental findings cost-effective?

## Ethical aspects

In seminal work on the validation of screening procedures, Cochrane and Holland mentioned: “We believe there is an ethical difference between everyday medical practice and screening. If a patient asks a medical practitioner for help, the doctor does the best he can. He is not responsible for defects in medical knowledge. If, however, the practitioner initiates screening procedures he is in a very different situation. He should, in our view, have conclusive evidence that screening can alter the natural history of disease in a significant proportion of those screened“.^35^ Furthermore, in landmark work on the principles and practice of screening for disease, Wilson and Jungner outlined 10 principles (unofficial preconditions) for screening (Table 1).^36^ Unrequested screening for incidental findings does not unequivocally comply with any of these 10 principles (Table 1).

**Table 1.** Ten principles (unofficial preconditions) for screening according to Wilson and Jungner,^36^ and compliance of unrequested screening for incidental findings with each of these principles

Principle	Compliance
The condition sought should be an important health problem	Unclear
There should be an accepted treatment for patients with recognized disease	Unclear
Facilities for diagnosis and treatment should be available	Unclear
There should be a recognizable latent or early symptomatic stage	Unclear
There should be a suitable test or examination	Unclear
The test should be acceptable to the population	Unclear
The natural history of the condition, including development from latent to declared disease, should be adequately understood	No
There should be an agreed policy on whom to treat as patients	No
The cost of case-finding (including diagnosis and treatment of patients diagnosed) should be economically balanced in relation to possible expenditure on medical care as a whole	No
Case-finding should be a continuing process and not a “once and for all” project	No

## Medicolegal aspects

To hold a physician liable for malpractice, a judge or jury must find that the physician’s conduct falls below the standard of care.^37^ The current standard of radiological care dictates that the entire imaging study should be evaluated, which includes a screening for incidental findings in all visualized organs. Interestingly, in a review about the medicolegal dilemma of incidental findings, it was stated that the question of whether to report or not report incidental findings is not easily answered.^38^ It is likely that specific situations in which radiologists over- or undercall incidental findings will generate occasional medical malpractice lawsuits, and it cannot be predicted how judges and juries will resolve such lawsuits.^38^

Because practicing diagnostic radiology with a closed Pandora’s box entails a deviation from the current standard of care, it could potentially lead to malpractice lawsuits. Legal and insurance experts may find this practice only acceptable if a very large study demonstrates that the benefit of non-detection of incidental findings improves patient’s health, and outweighs the advantage of early detection of some unexpected lesions. In other words, keeping Pandora’s box closed will only become medicolegally acceptable when it is considered standard of care.

Patients may also be involved in whether or not they want to be screened for incidental findings. If an incidental finding is regarded as a potential negative side-effect of imaging, and patients can be well informed, they may either choose to have their unmodified imaging examination screened for incidental findings or they may provide consent to keep Pandora’s box closed. Such an approach respects the autonomy of patients and may prevent malpractice lawsuits.

## Future directions

There is a lot of further work that needs to be done. First, segmentation algorithms to delete or selectively display organs on imaging studies need to be developed and optimized for clinical use. These algorithms should also be trained with data that include routine deviations from normal such as image artifacts, abnormal anatomy, and inserted materials such as catheters, drains, prostheses, and other devices, to ensure widespread clinical applicability of these algorithms. Second, future clinical studies can investigate if keeping Pandora’s box closed is cost-effective compared to the current standard of care. In a research setting, patients may be randomized in two arms, with one arm in which the imaging examination is not modified and another arm in which only clinically relevant organs are displayed and clinically irrelevant organs are deleted from the imaging examination. The main outcome measure will be the relative difference in quality-adjusted life years between the two arms. This analysis will have to be done for many different clinical scenarios and with long-term follow-up. Third, several workflow and governance issues have to be addressed. For example, it is currently unclear how an unmodified imaging study should be acquired and archived (and for how long), and meanwhile kept hidden from human eyes to avoid the accidental detection of an incidental finding. It should also be decided which persons are authorized to access or to grant access to the unmodified imaging study. Fourth, society has to reflect on, and eventually decide whether the standard of care should be untargeted screening for incidental findings or to keep Pandora’s box closed.

## Summary

Incidental findings are common. Once detected, they can usually not be ignored, although they generally result in low-value and potential harmful care. A new method to pre-emptively tackle incidental findings is by keeping the proverbial Pandora’s box closed, *i.e*. to not visualize them. This can be achieved by applying deep learning-based organ segmentation algorithms that eliminate incidental findings from medical images prior to diagnostic evaluation. Only clinically relevant organs will be shown on the imaging study to answer the referring physician’s clinical questions. This concept may potentially increase the value of diagnostic imaging studies and transform radiology practice (possible advantages for radiology practice are improved clinical reasoning and improved quality of imaging requests by referring physicians, and a faster image interpretation that is only focused on the organs that are clinically relevant). Incidental findings have been an accepted and sometimes even welcomed by-product of medical imaging. Changing this deeply rooted habit is a challenging task, but with education, financial incentives, further research, and rethinking established practice, keeping Pandora’s box closed may perhaps become the standard of care in the future.
